# The Impact of Entrepreneurship Education of Entrepreneurs on the Entrepreneurial Psychology of Sports Majors From the Perspective of Pedagogy

**DOI:** 10.3389/fpsyg.2021.727831

**Published:** 2021-12-16

**Authors:** Taofeng Liu, Mariusz Lipowski, Yingying Xue, Tao Xiao, Hongzhen Liu, Ruilin Xu, Kunpeng Liu, Zijian Zhao

**Affiliations:** ^1^Zhengzhou University Physical Education Institute, Zhengzhou, China; ^2^Department of Physical Education, Sangmyung University, Seoul, South Korea; ^3^Gdansk University of Physical Education and Sport, Gdansk, Poland; ^4^Yangzhou University Physical Education Institute, Yangzhou, China; ^5^Xinjiang Education Institute, Urumqi, China

**Keywords:** entrepreneurship education, data mining, entrepreneurial psychology, planning behavior, sport major

## Abstract

In recent years, with the continuous reform and innovation of the sports industry, the national training of sports talents has gradually developed into the training mode of skilled sports talents and professional talents in the field of sports. Therefore, the research on the influence of entrepreneurship education on the entrepreneurial psychology of sports majors has become the inevitable requirement of the development of the sports industry. The purposes are to understand the entrepreneurial psychology and its influencing factors of the students in sports majors after graduation and promote more suitable college students to start businesses and realize self-value. With the students in sports majors in four colleges of Y province as the research object, the typical model in psychology, planning behavior model, is taken as the basic theoretical basis. The questionnaire method combined with the data mining technology based on the decision tree model is adopted to study the influencing factors of entrepreneurial psychology of sports majors. It focuses on the influencing factors and mechanisms of the entrepreneurial drive of sports students. The results show that the three factors, namely, entrepreneurial behavior attitude, entrepreneurial subjective norms, and entrepreneurial perceptual behavior control, are different and interrelated. They are inseparable and can be transformed into each other under certain conditions. Three factors jointly drive the entrepreneurial behavior of students in sports majors. The entrepreneurial drive of students in sports majors in Y province is a dynamic system mechanism, which is analyzed using data mining technology. The entrepreneurial perceptual behavior control is the core factor affecting the entrepreneurial drive of students in sports majors. However, the success rate of entrepreneurs will be higher when the three elements play a reasonable role. The subjective factors driving their entrepreneurship will be reduced in direct proportion when entrepreneurs are deficient in one aspect.

## Introduction

The number of college students continues to grow with the expansion of college enrollment, and more people have received higher education. According to *The Statistical Bulletin on the Development of National Education in 2020* presented by the Ministry of Education, in 2020, colleges in China enrolled 1,106,600 postgraduates, 9.6745 million undergraduate and junior college students in general higher education, and 3.6376 million students in higher education. Moreover, the growth rate has been rising. The national quality of China has also been improved, but it also brings some practical problems. First, the number of college graduates is increasing, and the competition is becoming increasingly greater. Second, the pulling effect of the economic slowdown of China on employment is weakened, and college students will also be limited by their conditions such as lack of work experience. The current employment situation is not so optimistic under various pressures. Facing this employment situation, more and more college students realize that only with innovative ideas and innovative thinking can they be competitive in the increasingly fierce competition and rapidly changing world. Moreover, more and more college students begin to try to start their businesses. College students have also received the attention and practical support of the government, society, colleges, and other parties.

At present, there is a huge contradiction between the growing health needs of the people and the urgently needed professionals in sports. Meanwhile, the endowing status of sports talents by employers has put forward a severe test for the employment of sports college students who are about to graduate. The development of the sports industry and sports cause is inseparable from the cultivation of talents. However, at present, the cultivation of talents in the sports industry has obvious shortcomings when the development of the sports industry has entered the fast lane. The lack of talents has become one of the obstacles restricting the rapid development of the sports industry of China. According to the 13th five-year plan of the national sports industry, the employed population of the sports industry of China reached 6 million in 2020. At present, there are only hundreds of thousands of graduates trained by national sports colleges and non-sports colleges, so there is a large talent gap, let alone many sports graduates who are not engaged in sports-related industries. To realize the employment of college students in sports majors, the state continues to encourage them to start businesses. Hence, the research of entrepreneurship education on the entrepreneurial psychology of sports majors from the perspective of pedagogy is conducive to promoting the entrepreneurship of sports talents with outstanding professional quality and strong comprehensive ability. Besides, it can realize entrepreneurship to drive employment and then fully improve the quality of entrepreneurship and employment of sports majors. Moreover, it can break the shortage of employment in enterprises and the difficult employment of students, meet the health needs of people, and effectively improve the success rate of sports talents.

The research innovations are as follows. First, the knowledge of psychology, artificial intelligence, and deep learning are integrated across disciplines. Analyzing the driving factors of entrepreneurship of students in sports majors can make people deeply understand the influencing factors of their entrepreneurship psychology and provide sufficient theoretical guidance for the development of the sports industry and the cultivation of sports talents. Second, with the students in sports majors as the research object, the influencing factors of their entrepreneurial psychology are analyzed. It can make people more specifically understand and grasp the main influencing factors and specific reasons of their entrepreneurial psychology, constantly promote the employment of them by entrepreneurship, and promote the development of sports entrepreneurial talents in China. Third, the uniqueness of group entrepreneurship of college students in the process of model construction is highlighted, and the relevant influencing factors are analyzed from multiple dimensions.

## Literature Review

The relevant materials of China Academic Journal Network, Wanfang full-text database, and YALU Chinese Journal Network are considered based on the research purpose and content. Besides, they are analyzed and understood in detail by reading the related books of entrepreneurship of college students and entrepreneurship drive theory, to lay a good theoretical foundation for this exploration.

The entrepreneurship research status of students in sports majors is as follows. Adi et al. (2021) found that empowering leadership is positively correlated with the feedback of followers seeking in the innovation and development of enterprises; feedback-seeking of employees is positively related to task performance, responsible person, and right to speak, and it mediates the positive relationship between empowering leadership and task performance, as well as a responsible person and right to speak. Brieger et al. (2021) studied the individual psychological adaptation of entrepreneurs using a questionnaire. The results show that the challenges faced by entrepreneurs come from personal inner pressure, unknown environment, cultural differences, and so on. Excessive pressure will cause ideological and psychological burdens to entrepreneurs and even lead to physical symptoms. However, appropriate pressure can play a leading role in promoting the smooth progress of entrepreneurship work.

Chen (2019) studied the relationship between competition, admiration, and vulnerability. The results show that there is a positive correlation among them. Meanwhile, it supported the theory that vulnerability is negatively correlated with stability and plasticity, replicates the relationship between admiration and competition as well as personality traits and meta traits, and expands the existing knowledge. They also investigated the four satisfaction factors, namely, trust, profit, learning, and social interaction of network entrepreneurial groups. The results show that among the three kinds of social media (Line, Facebook, and Wechat), the structure and function of the four satisfaction factors are different. The two satisfaction factors, namely, trust and profit, can be regarded as the specific satisfaction of the network entrepreneurship group. In particular, the trust factor deserves more attention in the further research of social media network entrepreneurship courses. Du et al. (2021) explored and found the importance of the driving factors behind entrepreneurial intention (EI) on entrepreneurship education and entrepreneurship practice. The results show that the positive prediction of the dark triad has a partial mediating effect on the dark triad and EI.

Through investigation and research, Ghada (2021) suggested that whether an individual decides to start a business or not will be affected by the external entrepreneurial conditions and environment, and whether a business can succeed or not depends on the internal drive of the entrepreneur. Huang et al. (2021) found that the ability of learners to work cooperatively and coordinate efforts in a team becomes increasingly crucial for the success of any work and the progress of knowledge. Hu and Ye (2021) conducted multiple regression analyses on the survey data of 205 incubators in 14 Chinese business incubators. It was found that the internal and external network of business incubators positively affects the growth performance of enterprises, and exploratory and exploitative learning mediates the relationship between the two. To investigate the relationship between social capital and EI of college students and further investigate the intermediary effect of psychological capital between them, Qian et al. (2018) used social capital questionnaire, positive psychological capital scale, and EI scale to measure 594 college students.

Understanding the cognitive infrastructure supporting entrepreneurial activities can provide a richer perspective on how to cultivate entrepreneurship. The introduction of social cognitive theory in the field of entrepreneurship is conducive to understanding the process of entrepreneurs discovering and utilizing opportunities and exploring the essence of entrepreneurship. Zhang et al. (2019) proposed and tested a three intermediary effect model using the planned behavior theory. Among them, entrepreneurial learning was related to EI through attitude, subjective norms, and perceptual behavior control of entrepreneurship. Besides, the regulatory effect of previous exposure to entrepreneurship was proposed and tested. Moreover, based on a sample of 200 college students who have taken entrepreneurship courses in Hong Kong, hierarchical regression and adjustment intermediary tests were used to test the hypothesis. This exploration takes this as a reference to explore the impact of innovation and entrepreneurship education on the entrepreneurial psychology of students in sports majors.

Entrepreneurship education began in 1947 when Myles Mace offered the course “start-up business management” for MBA students of Harvard Business School. This course is considered to be the starting point of entrepreneurship education. Entrepreneurship education has two definitions: broad sense and narrow sense. The broad sense is to cultivate a pioneering spirit of students, which is a kind of quality education; the narrow sense is an education to train individuals to engage in industrial and commercial activities, train students from job seekers to job creators, provide jobs, and create jobs.

Thereby, entrepreneurship education is to focus more on cultivating the entrepreneurial consciousness of individuals engaged in enterprises and business planning, cultivating the entrepreneurial spirit such as pioneering spirit and adventure spirit, enhancing entrepreneurial knowledge and skills, improving the comprehensive quality of individual entrepreneurship, and providing preparation and guidance for the practice of individual independent entrepreneurship.

## Methods

### Driving Theory of Planned Behavior

#### Theoretical Model

**Figure 1:**
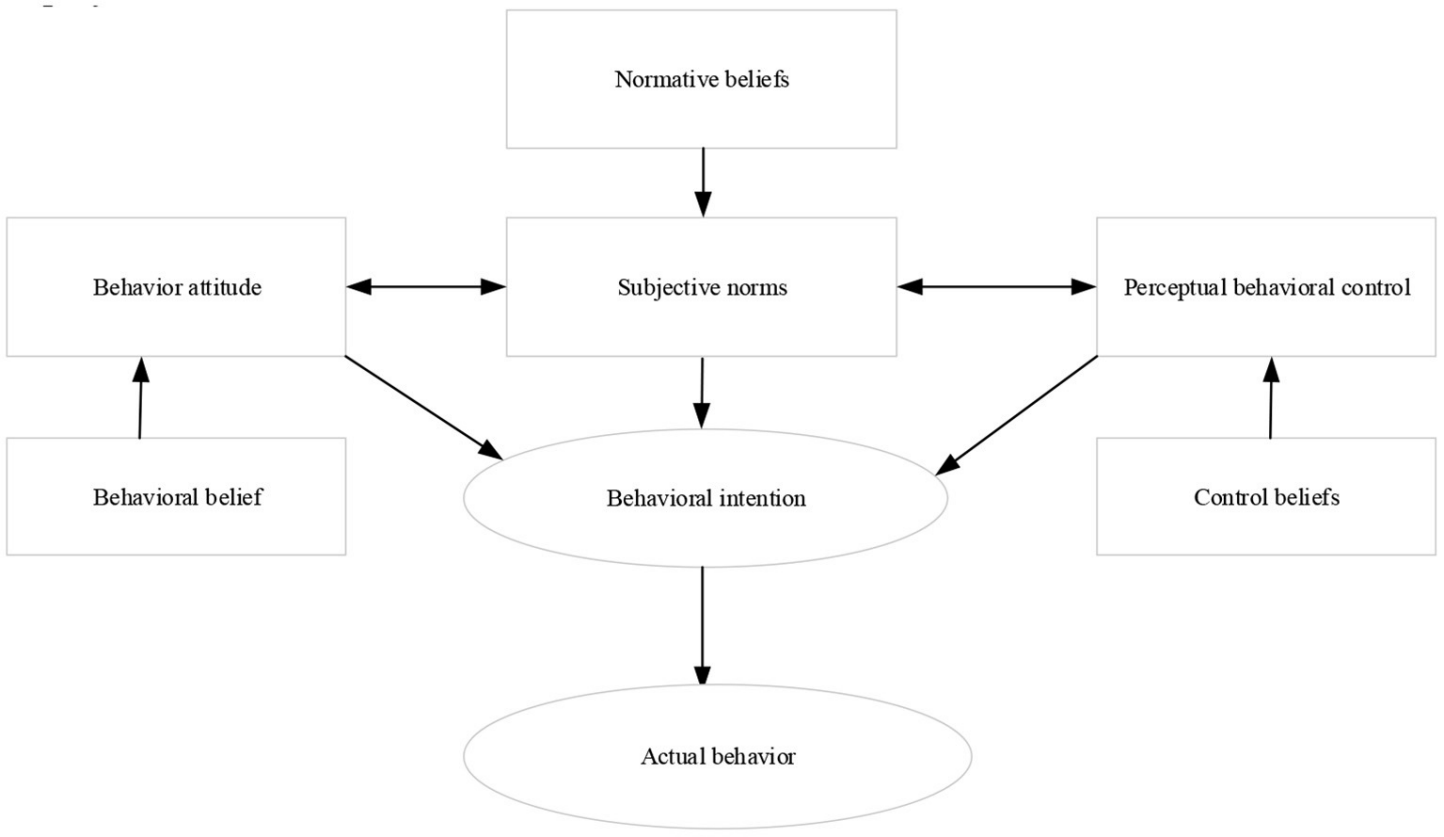
A theoretical model of planned behavior.

Koh (2021) suggested that the theoretical model of planned behavior was selected since it was one of the famous methods to demonstrate the relationship between consciousness and behavior in the field of social psychology. The entrepreneurial practice of entrepreneurs is predicted to explore the relationship between the entrepreneurial psychological factors and the entrepreneurial drive of sports majors. The application of this theory is due to its three dimensions, namely, individual behavior attitude, subjective norms, and perceptual behavior control, which work together to drive individual behavior. Figure 1 displays the theoretical model based on this.

Liu (2020) stated that the theoretical model described the relationship among behavior attitude, subjective norm, and perceptual behavior control. The attitude of an individual toward behavior results, perception of subjective norm, and perceptual behavior control determines the behavioral intention. The theory identifies three antecedent variables of behavioral intention construction. Liu and Yuan (2018) proposed that the first is the attitude toward the result of behavior, and the second is the perception of subjective norms. These two antecedent variables jointly reflect the demand for the perception of behavior. The third is behavior perception control, which is mainly reflected in the feasibility of individual behavior.

#### Research Variable Selection

Based on the above theoretical framework and some basic information required in the actual survey, the selection of research variables in the questionnaire mainly includes the following three parts.

The first part is to understand the basic information of the respondents; the second part is to understand the current situation of entrepreneurial driving, attitude, and motivation of the respondents; the third part is to further understand the information of the influencing factors of the entrepreneurial driving of the respondents, which mainly includes entrepreneurial behavior attitude factors of college students, perceptual behavior control, and subjective norms. The answers of the respondents to the relevant questions including entrepreneurial behavior attitude factors, perceptual behavior control, and subjective norms reveal the specific relationship of various factors affecting the entrepreneurial drive of college students in sports majors. Table 1 displays the details.

**Table 1:** Research variable selection.

**Entrepreneurial behavior attitude**	**Entrepreneurial perceptual behavior control**	**Entrepreneurial subjective norms**
Sense of worth	Innovation ability	Project opportunity
Moral concepts	Social distribution ability	Family factors
Will quality	Management	School factors
Faith and determination	Teamwork	Social atmosphere
Cognitive structure	Market foresight ability	Macro policy
Intention and vision	Distribution of fund-raising capacity	Market environment
Hobby		
Temper and character		

#### Data Mining Method Based on Decision Tree

**Figure 2:**
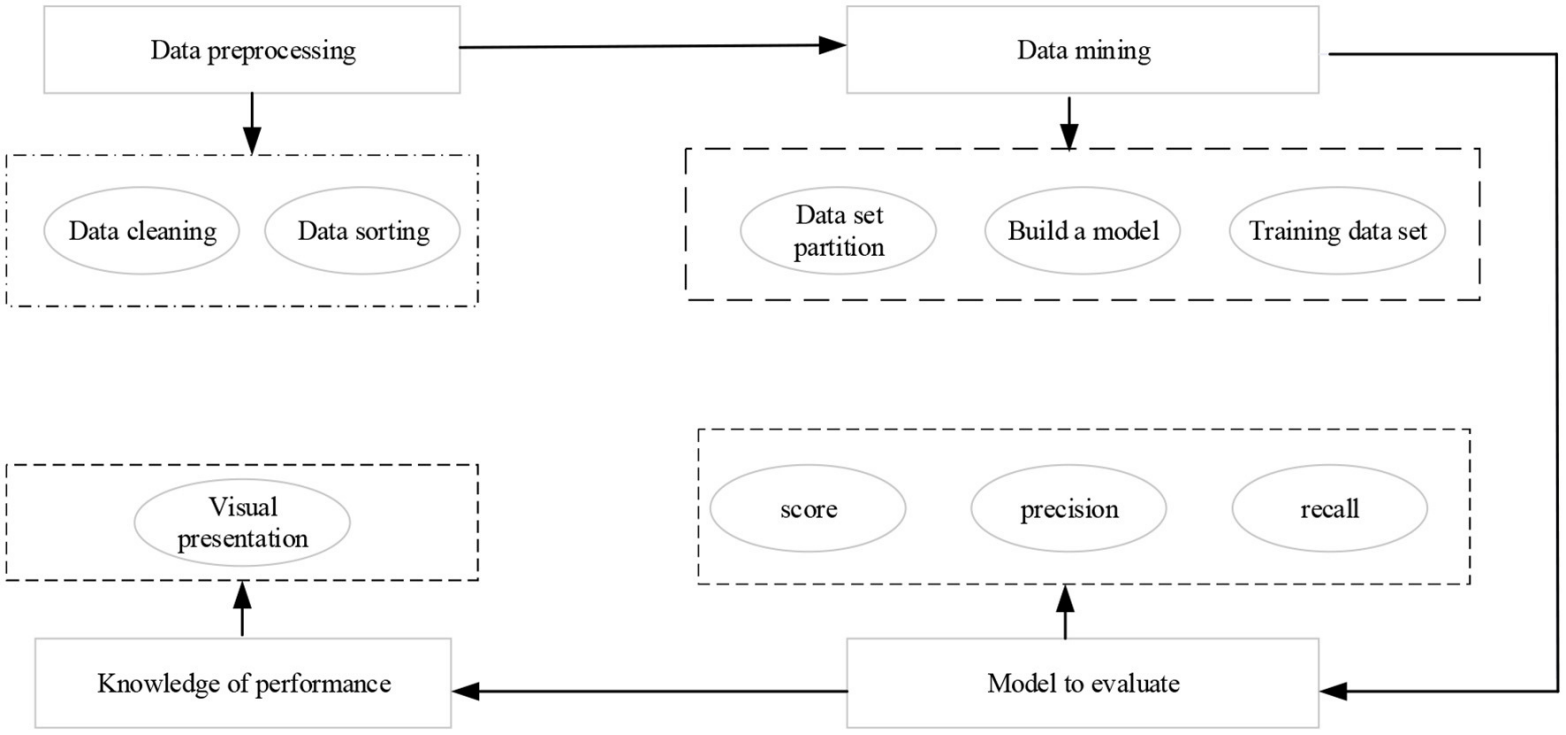
Decision tree model of data mining.

Ma et al. (2020) suggested that data mining technology is extended to multiple fields with the continuous improvement of science and technology. People calculate and process massive data through data mining to get the required data model, through which the research results can be presented more intuitively and daily problems can be solved. Manea et al. (2019) indicated that the data mining technology was introduced into a deep analysis of the statistical results of the questionnaire to get the influence mechanism of an entrepreneurial drive of the sports students. Figure 2 is the data mining model of the decision tree. Mcmullen et al. (2020) stated that the CART algorithm is adopted to mine the data of entrepreneurship questionnaire of college students in sports majors, and the decision tree model is obtained. The generated decision tree is traversed, and each path from the heel node to the leaf node corresponds to a rule for rational analysis.

### Research Objects

According to the research purpose and content needs, 180 undergraduates and graduates in sports majors from 2016 to 2020 in two colleges in Y province and two colleges in S province were selected as the research objects to mainly explore the driving factors of entrepreneurship.

### Research Method

#### Documentation Method

Based on the research purpose and content, the relevant materials, such as China Academic Journal Network, Wanfang full-text database, and YALU Chinese Journal Network, were considered. Among them, there were 4 dissertations, 10 journal theses, 5 relevant policy documents, and 15 books on entrepreneurship and entrepreneurship driving theory of college students in sports majors. These materials were carefully analyzed and understood to lay a theoretical foundation for this exploration.

#### Questionnaire Method

This exploration was mainly to discuss the impact of entrepreneurship education on the entrepreneurial drive of sports majors. Hence, a questionnaire method was employed to study the entrepreneurial driving factors of college students in sports colleges and departments of Y province. In the second half of 2020, designed questionnaires were randomly distributed to students from two colleges in Y province and two colleges in S province. Then, 200 questionnaires were collected from graduates on the Internet. Among them, 190 questionnaires were successfully recovered, and the recovery rate was 95%; there were 180 valid questionnaires, and the effective rate was 94.7%. Table 2 displays the specific research contents.

**Table 2:** Questionnaire of entrepreneurial driving factors of college students in sports majors in Y province.

**Research questions**	**Options**
What do you think of your three views?	A: Very inconsistent B: inconsistent C: no idea D: consistent E: very consistent
What do you think of your understanding of entrepreneurship?	A: very poor B: poor C: general D: good E: very good
What do you think of your will quality?	A: very poor B: poor C: general D: good E: very good
What do you think of your temper and character?	A: very poor B: poor C: general D: good E: very good
What do you think of your faith?	A: very poor B: poor C: general D: good E: very good
What do you think of your vision planning for entrepreneurship?	A: very poor B: poor C: general D: good E: very good
What do you think of your innovation ability?	A: very poor B: poor C: general D: good E: very good
What do you think of your social skills?	A: very poor B: poor C: general D: good E: very good
What do you think of your management ability?	A: very poor B: poor C: general D: good E: very good
What do you think of your teamwork skills?	A: very poor B: poor C: general D: good E: very good
What do you think of your market foresight?	A: very poor B: poor C: general D: good E: very good
If you start a business, what do you think of? your ability to raise funds?	A: very poor B: poor C: general D: good E: very good
If you start a business, what will be the attitude of your family and friends?	A: firmly against B: against C: unknown D: support E: firmly support
If you start a business, what do you think of the attitude of the public?	A: serious incomprehension B: incomprehension C: unknown D: appreciation E: high appreciation
What do you think of the praise of the mainstream media in Y province for the typical entrepreneurship?	A: very poor B: poor C: general D: good E: very good
How do you evaluate the service consciousness of the government and the college for college students' entrepreneurship?	A: very poor B: poor C: general D: good E: very good
What do you think of the current government policies and measures for college students' entrepreneurship?	A: very poor B: poor C: general D: good E: very good
What do you think of the support of government projects in Y province for college students' entrepreneurship?	A: very poor B: poor C: general D: good E: very good

#### Data Analysis Method

SPSS18.0 statistical software (Chicago: SPSS Inc.) of social sciences was adopted. The questionnaires were distributed, and the effective questionnaires were successfully recovered for necessary mathematical statistics and result analysis.

In Table 3 Six experts were selected to make the research data and questionnaire design more reliable, among which three were professors (one was engaged in career guidance, one was the leader of the incubator, and one was the entrepreneur). The validity of the questionnaire is tested by filling in the questionnaire and interviewing. Overall, 6 questionnaires were distributed and successfully recovered, with an effective rate of 100%.

**Table 3:** Questionnaire validity scale.

**Validity**	**Very**	**Basically**	**Effective**	**Less**	**Invalid**
	**effective**	**effective**		**effective**	
Overall design	1	4	1	0	0
Content design	0	3	3	0	0
Structural design	2	1	3	0	0

A total of 20 influencing factors of the entrepreneurial drive were randomly selected as indexes from the three dimensions of the theory of planned behavior. The total score of this questionnaire was 100 points, and the corresponding scores of each dimension were 35, 35, and 30 points, respectively. The Likert-type 5-level scoring method was adopted, with a median of 3.

### Dimensions of Entrepreneurial Drive

**Figure 3:**
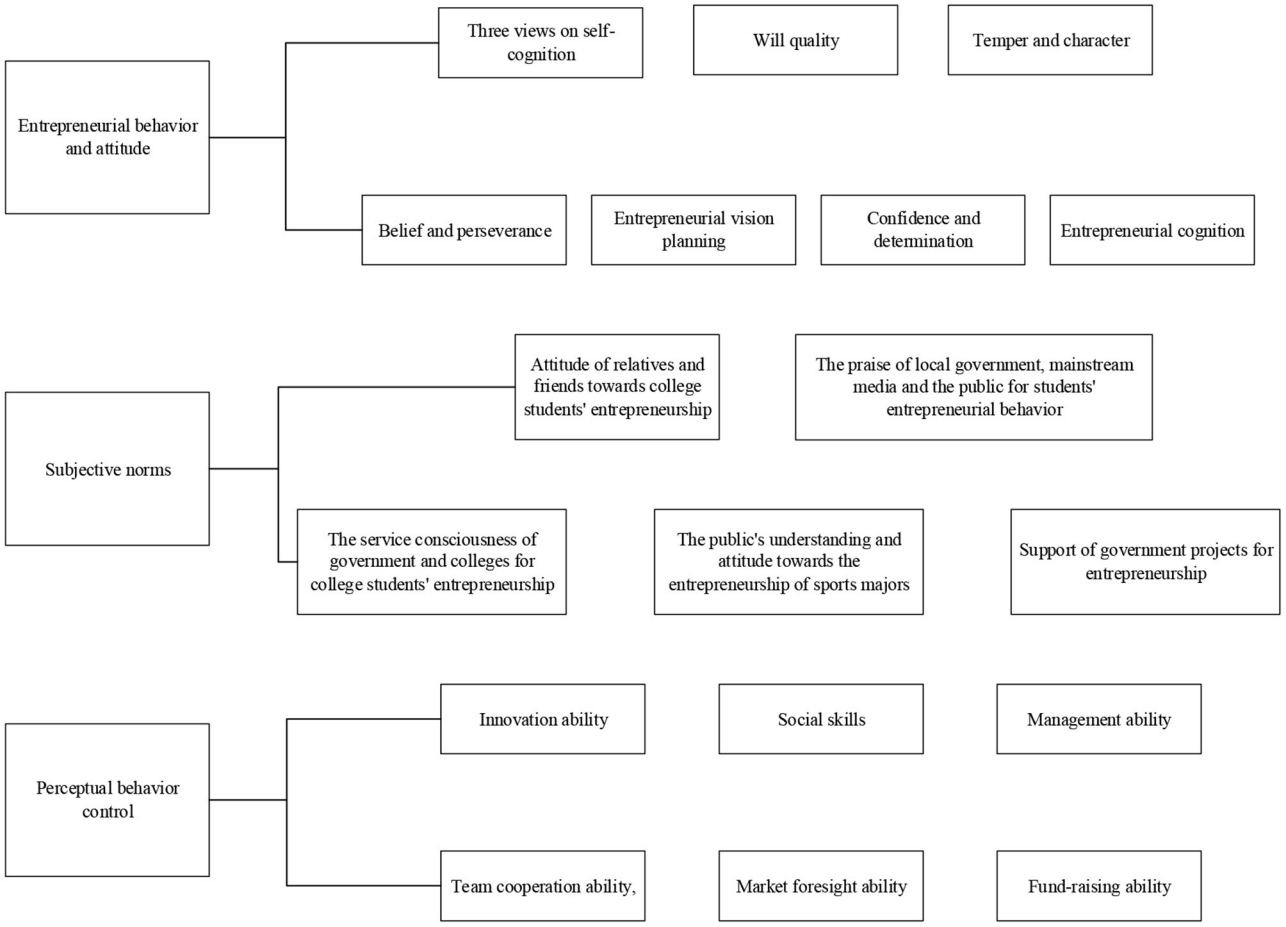
Dimension map of entrepreneurial drive.

The actual situation of entrepreneurial drive of sports students includes three dimensions: entrepreneurial behavior attitude, subjective norm, and perceptual behavior control. Figure 3 is the statistics of different dimensions controlled by it.

## Research Results and Analysis

### An Analysis of the Status Quo of Entrepreneurial Behavior Attitude of Sports Majors

**Figure 4:**
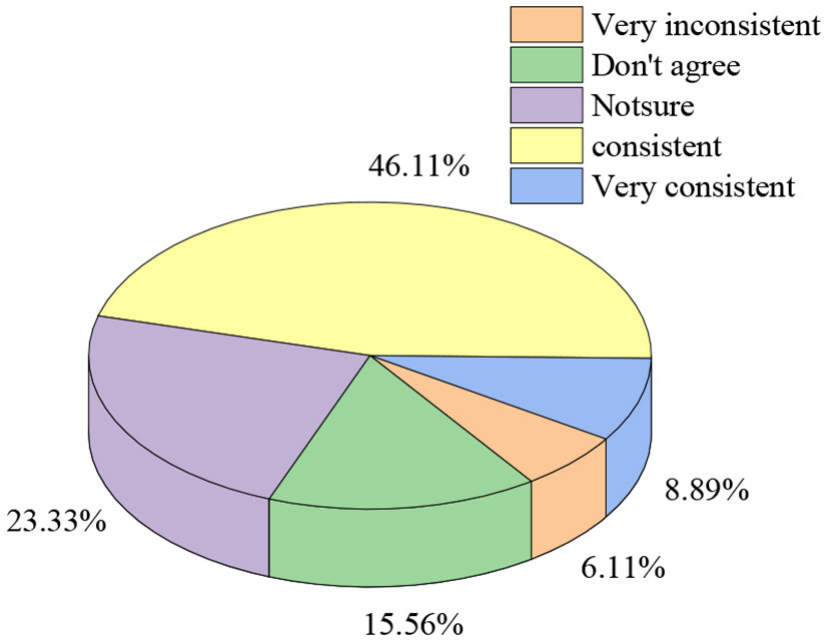
Distribution of three views and self-cognition.

**Figure 5:**
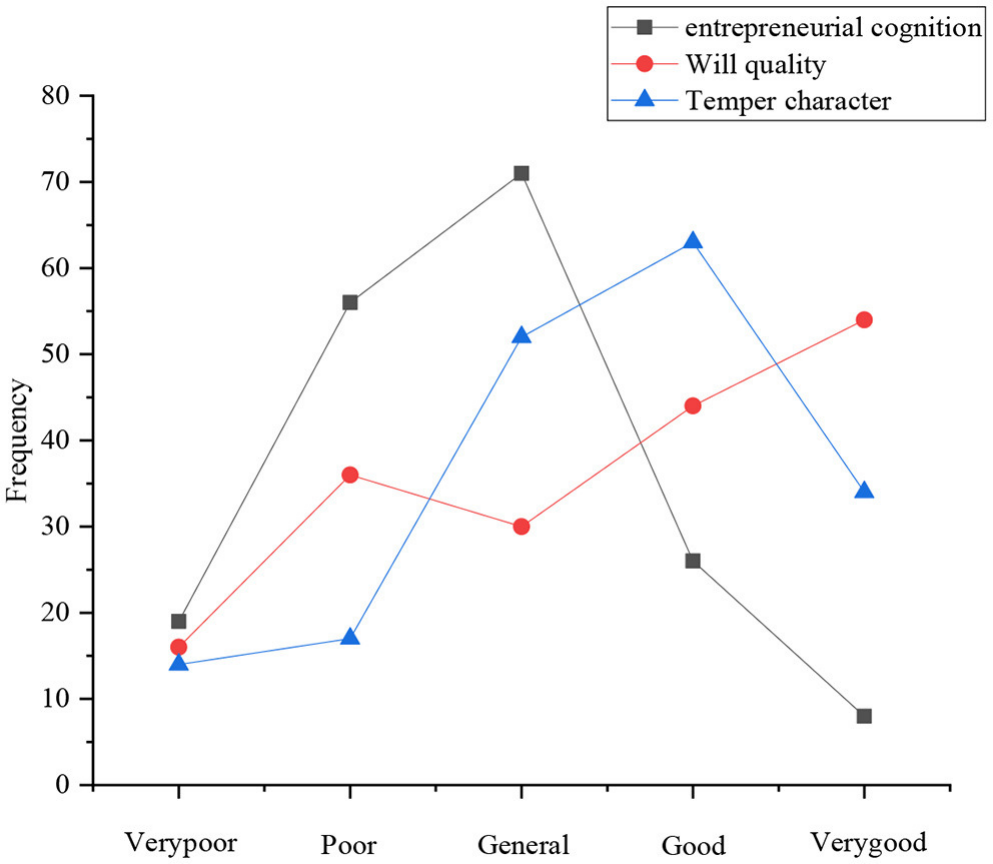
Distribution of entrepreneurial cognition, will quality, and temper character.

The investigation of entrepreneurial behavior attitude of sports majors involves seven aspects: three views and self-cognition, entrepreneurial cognition, will quality, temper and character, belief and perseverance, entrepreneurial vision planning, and confidence and determination. Figure 4 shows the statistical results of the consistency of the three views and self-cognition. Figures 4, 5 present the other statistical results.

Figure 4 displays that regarding the choice of three views and self-cognition, respondents who choose “very inconsistent,” “don't agree,” “not sure,” “consistent,” and “very consistent” account for 6.11, 15.56, 23.33, 46.11, and 8.89%, respectively. The data show that the overall three views and self-cognition of the current sports majors are relatively consistent.

Figure 5 reveals the entrepreneurial cognition structure of college students in sports majors. The number of students who choose general, poor, good, very poor, and very good is 72, 57, 25, 19, and 9, respectively. Statistics show that the cognition structure of the current sports college students of entrepreneurship is still relatively lacking and needs to be strengthened. Regarding the will quality, the number of students who choose very good, good, poor, general, and very poor is 54, 41, 37, 30, and 16, respectively. It suggests that nearly half of the students think that they are better in will quality, and less than half of the students think that their will quality needs to be strengthened. Mu et al. (2020) showed that in the evaluation of their temper and character, the number of students who choose good, general, very good, poor, and very poor is 63, 52, 35, 16, and 13, respectively. Raza et al. (2021) indicated that the vast majority of college students think that they have an ordinary or better temper and character, and only a few of the students think that their temper and character are very bad.

**Figure 6:**
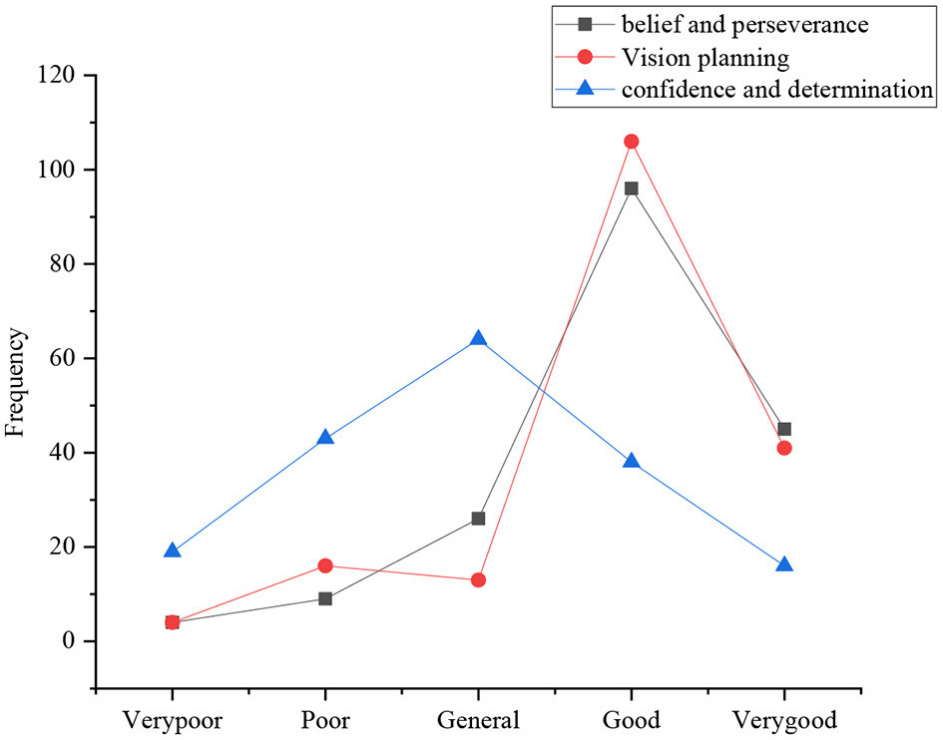
Distribution of belief and perseverance, vision planning, and confidence and determination.

Figure 6 displays the evaluation of students of confidence and determination for future entrepreneurship. The number of students who choose good, very good, general, poor, and very poor is 98, 50, 22, 9, and 5, respectively. It illustrates that the vast majority of college students think that they have a very firm belief and perseverance, and a few students are afraid that they cannot stick to it; regarding their future entrepreneurial design vision and planning, the number of students who choose good, very good, poor, general, and very poor is 108, 39, 18, 11, and 4, respectively. Statistics show that the vast majority of college students think that they will plan their future business vision and planning; in the evaluation of their confidence and determination to start a business in the future, the number of students who choose general, poor, good, very poor and very good is 62, 41, 38, 20, and 12, respectively. It suggests that the confidence and determination of most of the students in entrepreneurship need to be strengthened, while only a few of them are very confident and determined.

Figures 4–6 illustrate that the three views and self-cognition of sports majors in Y province are relatively consistent, and they lack the cognitive structure needed for entrepreneurship. They all think that they have a goodwill quality as well as temper and character. They have the consideration of early planning and good vision in entrepreneurship, while their confidence in entrepreneurship needs to be strengthened.

### The Current Situation of Entrepreneurial Perceptual Behavior Control

**Figure 7:**
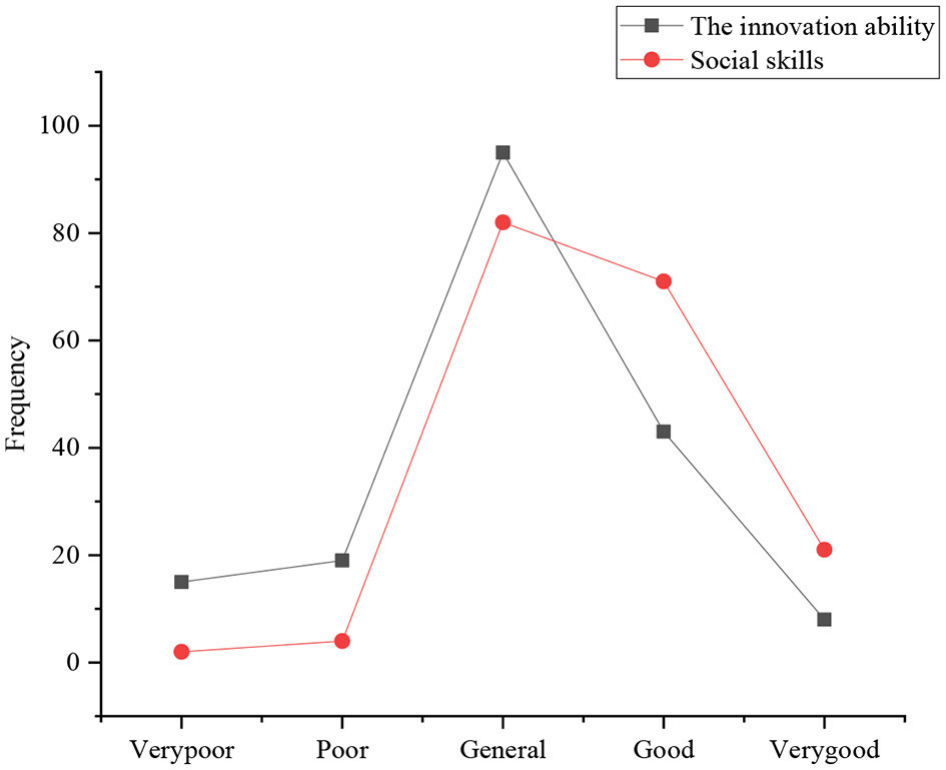
Distribution of innovation ability and social skills.

**Figure 8:**
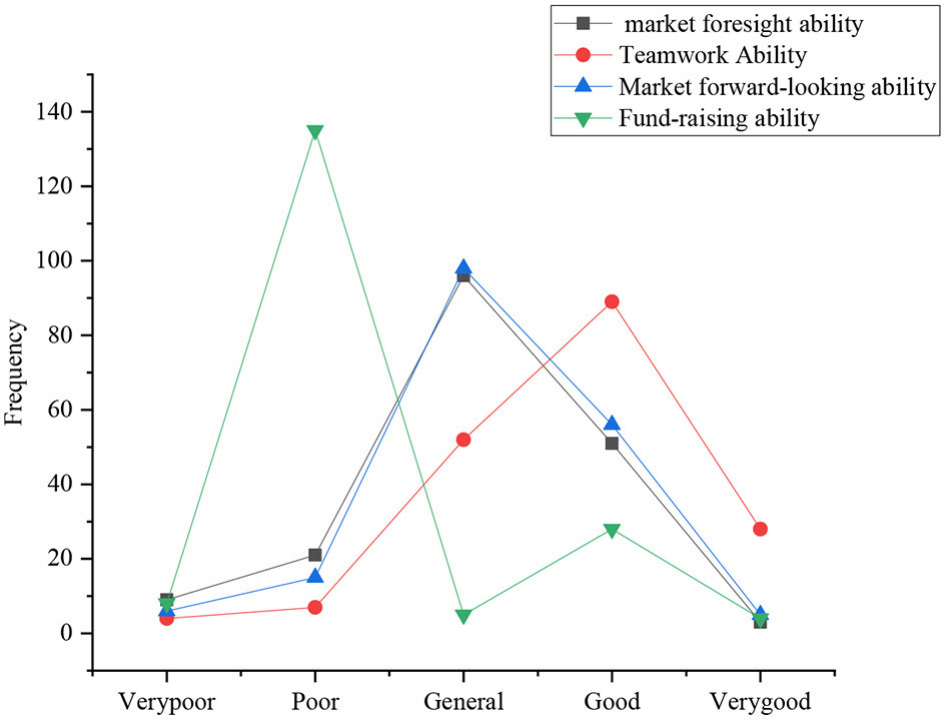
Distribution of business management, teamwork, market foresight, and fund-raising ability.

The investigation of behavior control of the sports students involves six aspects: innovation ability, social skills, management ability, team cooperation ability, market foresight ability, and fund-raising ability. Figures 7, 8 present the statistical results.

Rivas and Espada (2021) suggested that regarding the evaluation of their innovation ability, the number of students who choose general, good, poor, very poor, and very good is 97, 40, 20, 17, and 9, respectively. It reveals that most of the current college students are not satisfied with their innovation ability, even very pessimistic; only a few students think that they have a certain innovation ability; in the evaluation of their social ability, the number of students who choose general, good, very good, poor, and very poor is 82, 70, 21, 3, and 1, respectively. It reveals that the current college students are very confident in their social ability.

Regarding the evaluation of their management ability, the number of students who choose general, good, poor, very poor, and very good is 97, 50, 20, 10, and 2, respectively. It clearly suggests that the evaluation of the current college students of their management ability is basically above average; regarding team cooperation ability, the number of students who choose good, general, very good, poor, and very poor is 90, 50, 28, 5, and 2, respectively. It suggests that the evaluation of the current college students of their team cooperation ability is basically above average, and they are more confident; for market foresight ability, the number of students who choose general, good, poor, very poor, and very good is 97, 59, 12, 3, and 2, respectively. Obviously, the current college students can identify with their market foresight ability, and about half of them have strong market foresight ability; for fund-raising ability, the number of students who choose poor, good, very poor, general, and very good is 135, 22, 3, 2, and 2, respectively. It illustrates that one of the main obstacles faced by college students is to raise venture capital, and 80% of the students think it is difficult to raise funds.

Rogoza and Zemojtel-Piotrowska (2018) suggested that the statistical results of Figures 6, 7 suggest that sports majors in Y province are not much satisfied with their innovation ability, even very pessimistic, and only a few students think that they have a certain innovation ability. Students are basically confident in their social skills and teamwork ability. Sergent et al. (2020) showed that the abilities of business management and market foresight are weak, basically in the lower middle level. Sturm et al. (2021) revealed that most of the respondents think it is difficult to raise venture capital, indicating that raising venture capital has been one of the most crucial obstacles to drive entrepreneurship.

### The Current Situation of Entrepreneurial Subjective Norms

**Figure 9:**
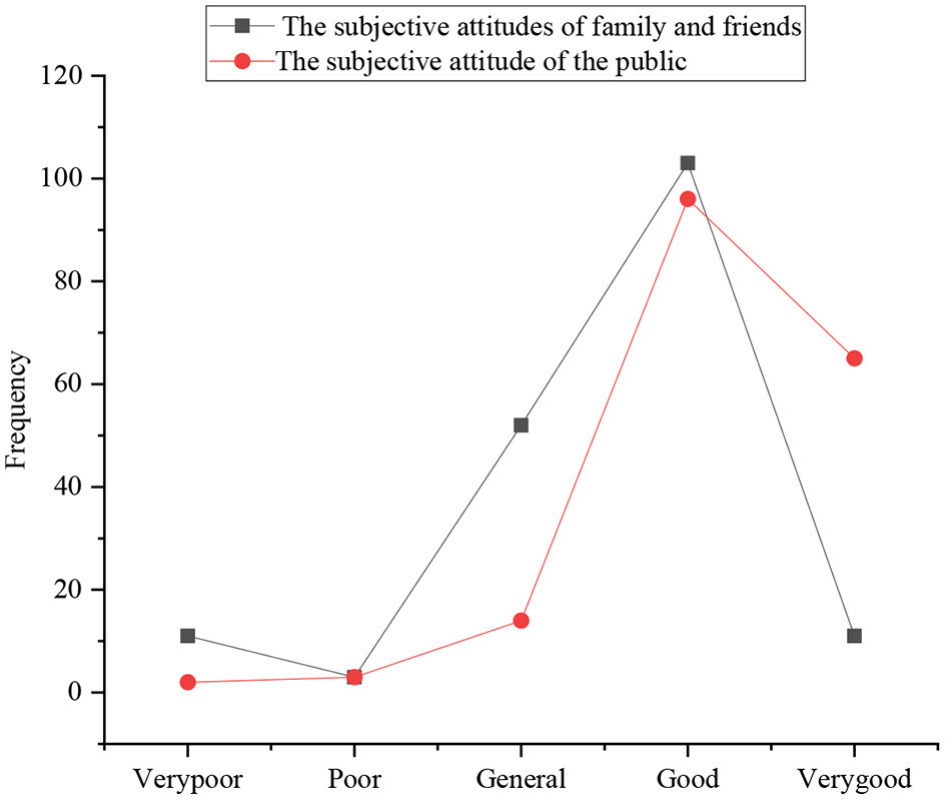
The distribution of subjective attitudes of family, friends, and the public toward the entrepreneurship of sports majors.

**Figure 10:**
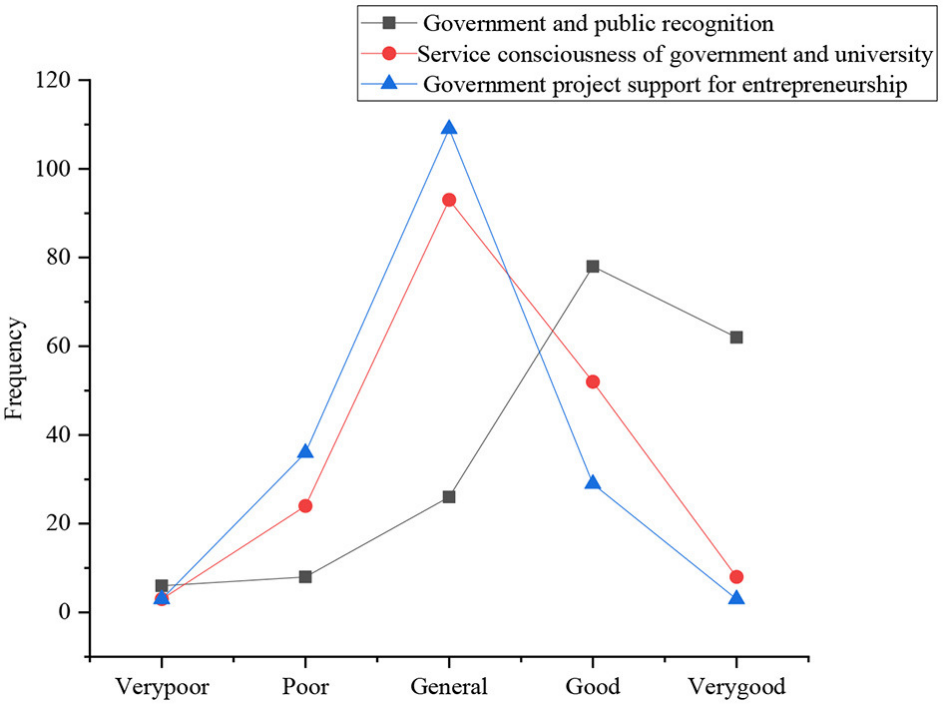
The distribution of government and public praise, service awareness, and project support for entrepreneurship of the sports majors.

The investigation of the subjective norms of sports majors involves five aspects: the subjective attitude of their relatives and friends toward their entrepreneurship, the attitude of the public toward entrepreneurship of sports majors, the praise of local government, mainstream media and the public for entrepreneurship of students, the service awareness of government and colleges for entrepreneurship of college students, and the support of government projects for entrepreneurship. Figures 9, 10 display the statistical results.

Regarding the attitude of the family and friends toward entrepreneurship of college students, the number of students who choose good, general, very poor, very good, and poor is 102, 50, 10, 9, and 2, respectively. It suggests that the respondents think that their family members and friends around them still show positive and supportive attitude toward entrepreneurship; in the evaluation of their fund-raising ability, the number of students who choose good, very good, general, poor, and very poor is 96, 68, 13, 2, and 1, respectively. Wang and Chen (2018) indicated that the current society and the public are still looking forward to the entrepreneurship of college students.

The government, the media, and the public praise the typical entrepreneurial behavior. As for their fund-raising ability, the number of students who choose good, very good, general, poor, and very poor is 79, 62, 22, 9, and 8, respectively. It suggests that they praise the typical entrepreneurial behavior positively; for the service consciousness of the government and colleges for entrepreneurship of college students, the number of students who choose general, good, poor, very good, and very poor is 92, 50, 23, 7, and 6, respectively (Wen and Hong, 2019). The figure shows that the current relevant government and colleges have a good evaluation on the service guidance of entrepreneurship of college students, which is in the upper level; for the support of the government projects for entrepreneurship, the number of students who choose general, poor, good, very good, and very poor is 110, 37, 25, 9, and 6, respectively. It suggests that government projects have a high degree of support for the entrepreneurship of college students.

Figures 8, 9 show that the surveyed college students think that their family and friends, the public, the relevant government, and the media understand and support their entrepreneurship on the whole, and the degree of praise is also very high. However, few college students choose to start a business, and the overall entrepreneurial atmosphere is not strong.

### Analysis on the Influencing Factors of the Entrepreneurial Drive of Students in Sports Majors

#### The Influence Mechanism of Various Factors on the Entrepreneurial Drive of the Sports Students

**Figure 11:**
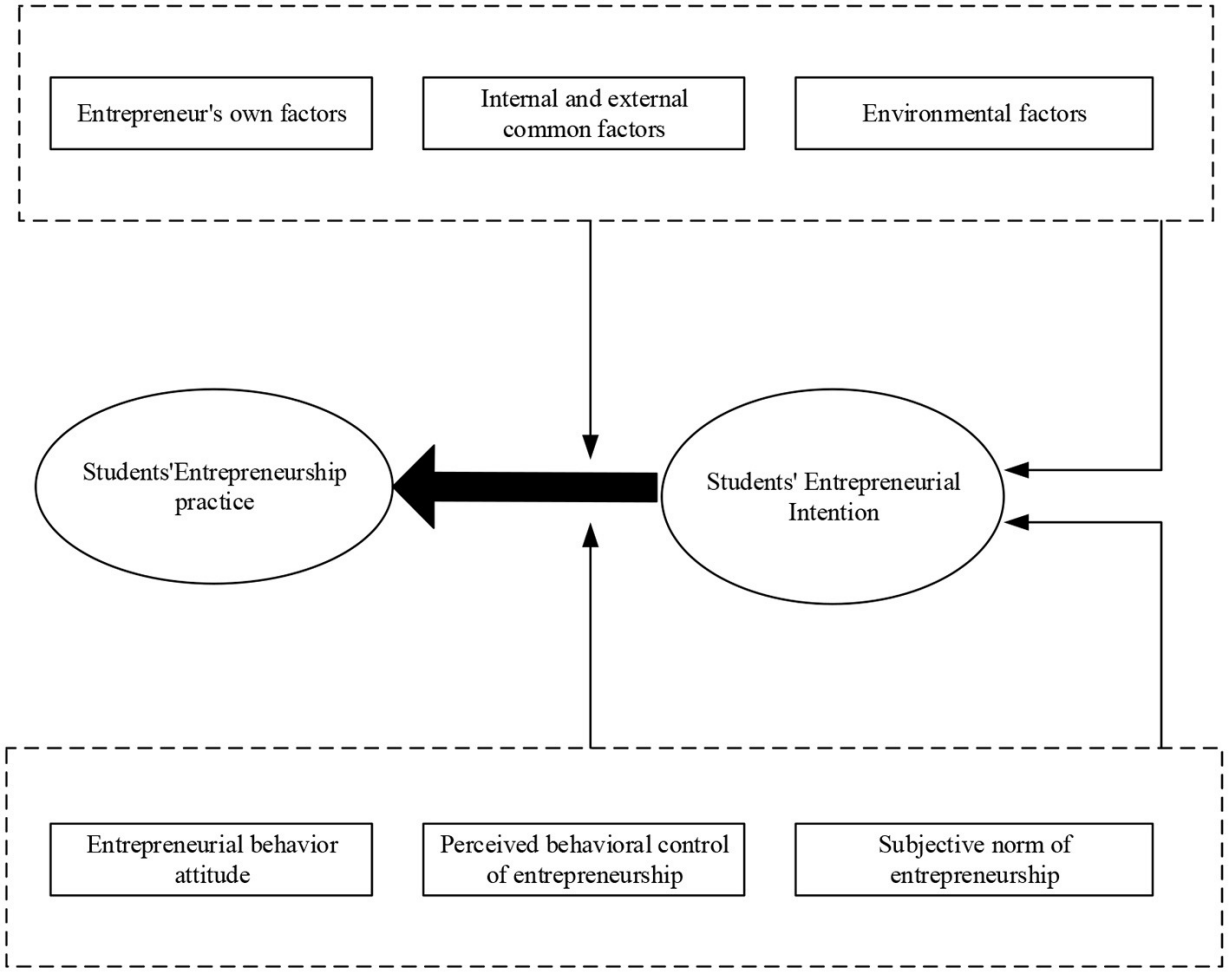
The influence mechanism of different factors on the entrepreneurial drive of college students in sports majors.

According to the survey results, the research on the entrepreneurial psychological mechanism is divided into three levels: entrepreneurial behavior attitude, perceptual behavior control, and subjective norms. Wenting (2021) proposed the dynamic mechanism of influencing factors of the entrepreneurial drive of sports majors. The internal driving force of entrepreneurs mainly includes entrepreneurial behavior attitude, the external driving force of entrepreneurs mainly includes entrepreneurial subjective norms, and the perceptual behavior control of entrepreneurs is a factor affected by both internal and external driving forces. Entrepreneurial behavior attitude, perceptual behavior control, and subjective norms jointly affect the entrepreneurial drive of college students in sports majors (Wen and Hong, 2019). Entrepreneurial perceptual behavior control can be directly transformed into entrepreneurial drive and then directly into entrepreneurial practice. Figure 11 displays the influence mechanism of different factors on the entrepreneurial psychology of college students in sports majors.

#### The Dynamic Mechanism of the Entrepreneurial Drive of Sports College Students

**Figure 12:**
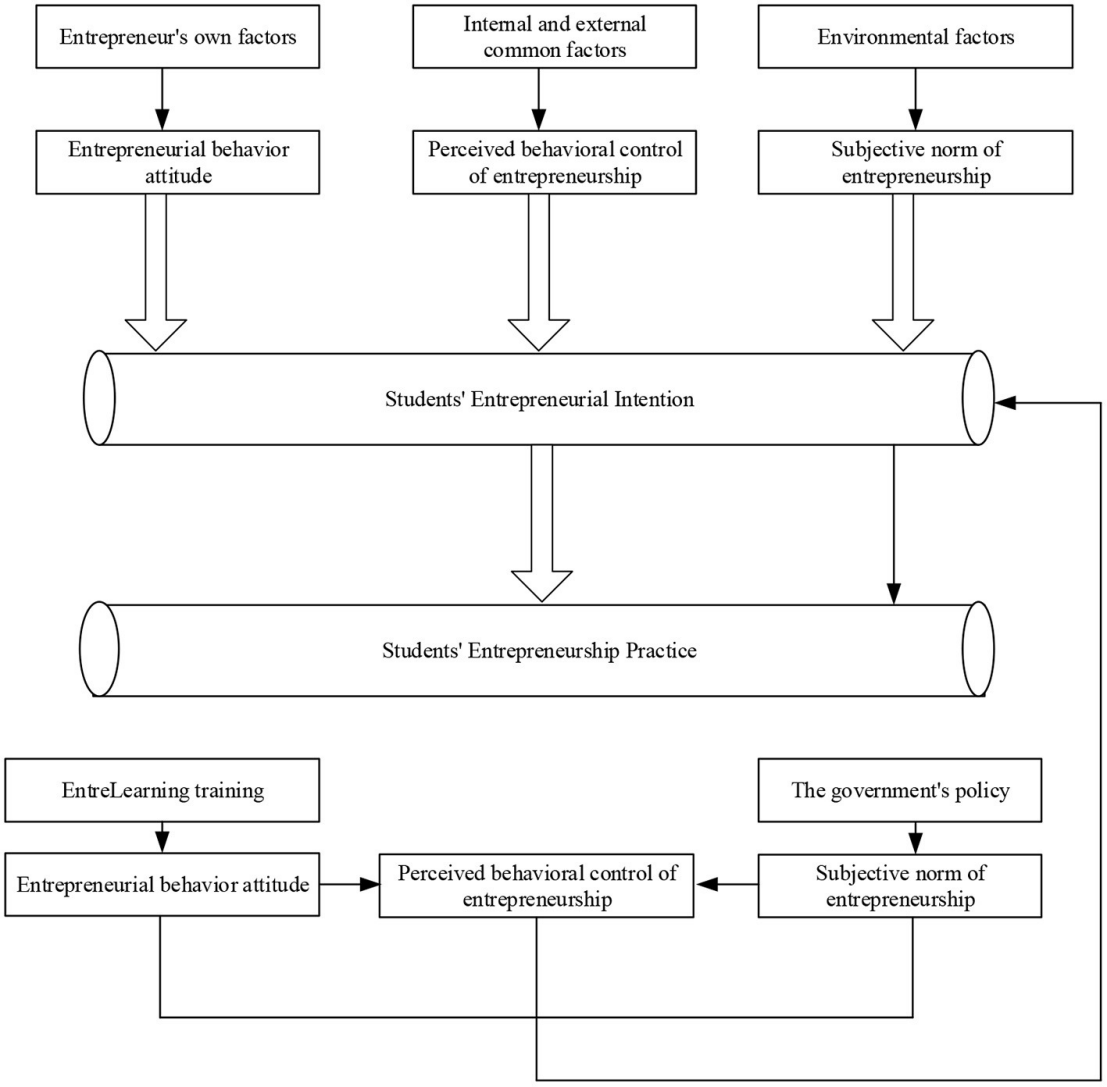
The dynamic mechanism of the entrepreneurial drive of the sports college students.

The data mining model constructed above is employed to analyze the survey results. Then, the dynamic mechanism of the entrepreneurial drive of sports majors in Y province is obtained, as shown in Figure 12.

The analysis of the dynamic mechanism of entrepreneurial drive in sports college students is as follows. Whether the college students are driven to conduct entrepreneurship practice or not depends on the rational analysis of the individual in the entrepreneurial environment, the recognition of the market, and the perceptual subjective psychological state of the demand. The three factors, namely, attitude, subjective norm, and perceptual behavior control, are different and interrelated. Among them, the entrepreneurial perception behavior control is the core factor affecting the entrepreneurial drive of college students in sports majors, which can directly drive entrepreneurs to carry out entrepreneurial practice activities. However, Wu and Song (2019) showed that the success rate of entrepreneurs will be higher when the three elements play a reasonable role in the individual entrepreneurs. The subjective factors driving entrepreneurship of the entrepreneurs will be reduced when they lack any factor, and *vice versa*. The three factors are inseparable and jointly drive the entrepreneurial behavior of college students in sports majors. Moreover, they can be transformed into each other under certain conditions. The entrepreneurial drive of sports majors in Y province is a dynamic system mechanism.

#### Discussion

The entrepreneurial practice of sports majors can react to the entrepreneurial drive (entrepreneurial behavior attitude, entrepreneurial perceptual behavior control, and entrepreneurial subjective norms), which has a positive proportional linear relationship. The optimization of the entrepreneurial atmosphere will bring a group effect with the strengthening of entrepreneurial motivation of college students, which will raise the entrepreneurial enthusiasm of the students in sports majors (Yuan and Wu, 2020). Through entrepreneurial practice, Zhao et al. (2020) found that the recognition of entrepreneurial behavior attitude, the accumulation of entrepreneurial behavior control, and the continuous strengthening of entrepreneurial subjective norms promote entrepreneurs to continue to learn and improve. To meet the market demand, it is required that government policy supply should be timely and effective, and there should be neither “vacancy” nor “dislocation,” to form a virtuous circle interaction between entrepreneurial conditions and entrepreneurial atmosphere (Zheng et al., 2018). Besides, in the process of writing a business plan and engaging in business practice, sports majors can enhance their ability of entrepreneurship and innovation, social skills, marketing ability, team management ability, cooperation ability, and market foresight ability. Of course, sports majors can also improve their entrepreneurial ability by participating in various forms of education and training related to entrepreneurship and innovation, which is particularly crucial to stimulate the entrepreneurial enthusiasm of sports majors (Zijun, 2021).

New ideas and understanding of entrepreneurial practice have been formed when entrepreneurial behavior attitude, perceptual behavior control, and subjective norms influence the entrepreneurial drive dynamics of sports majors. The newly formed entrepreneurial behavior attitude, perceptual behavior control, and subjective norms will further form a new impact on the entrepreneurial drive and entrepreneurial practice of sports majors, and form a dynamic closed-loop interaction.

## Conclusion

The survey results of entrepreneurial behavior attitude show that the three views and self-cognition of college students in sports majors in the two provinces are relatively consistent. They lack the cognitive structure required for entrepreneurship, and all think they have goodwill quality and temperament. Moreover, they have the consideration of advance planning and good vision in entrepreneurship, but their confidence in entrepreneurship needs to be strengthened; the survey of entrepreneurial perceived behavior control shows that raising entrepreneurial funds has been one of the main obstacles to entrepreneurship; the survey results of entrepreneurial subjective norms show that students in sports majors believe that their family and friends, the public, relevant governments, and media understand and support their entrepreneurial attitude and service consciousness as a whole, and their praise is also very high. However, few of the surveyed college students begin to try entrepreneurship, and the overall entrepreneurial atmosphere is not strong.

The three factors, namely, entrepreneurial behavior attitude, subjective norms, and perceptual behavior control, differ from each other and are interrelated. They are inseparable and can be transformed into each other under certain conditions. Entrepreneurial perceptual behavior control can directly drive entrepreneurial practice. The three factors jointly drive the entrepreneurial behavior of sports majors, which is a dynamic system mechanism for sports majors in Y province.

The research innovation is that the model highlights the uniqueness of the group entrepreneurship of college students. Unlike the independent entrepreneurship of the social group, entrepreneurship of college students has its uniqueness, which is fully considered in the process of model construction. Especially in the selection of dimensions affecting entrepreneurial tendency of college students, it is realized in combination with the particularity of identity of college students, making the research results more targeted and providing a certain reference for colleges to cultivate entrepreneurial tendency of college students; the influencing factors are analyzed from multiple dimensions. The research process is mainly around the dimensions of entrepreneurial behavior attitude, subjective norms, and perceptual behavior control. An in-depth analysis of the correlation between them and entrepreneurial drive is made.

The research limitations are that the amount of data is not enough and the scope is not wide enough. When a questionnaire on the entrepreneurial drive of college students in sports majors is conducted in these two provinces, the problems involved are not sufficient and cannot fully and accurately reflect their actual ideas, which still needs a lot of improvement and further analysis. Meanwhile, the difference of entrepreneurial drive between sports majors and other majors has not been studied, which can be the focus of future research to make the influence of entrepreneurial factors of college students more comprehensive and in-depth.

## Data Availability Statement

The raw data supporting the conclusions of this article will be made available by the authors, without undue reservation.

## Ethics Statement

The studies involving human participants were reviewed and approved by Zhengzhou University Ethics Committee. The patients/participants provided their written informed consent to participate in this study. Written informed consent was obtained from the individual(s) for the publication of any potentially identifiable images or data included in this article.

## Author Contributions

All authors listed have made a substantial, direct, and intellectual contribution to the work and approved it for publication.

## Funding

This work was supported by Research on the Governance of Henan Province Youth Extracurricular Traning Institutions in the New Era Project approval number: 2021YB0011 and Research on the Educational Standard System of Outstanding Physical Education Teachers in the New Era Project approval number: 21ATY009.

## Conflict of Interest

The authors declare that the research was conducted in the absence of any commercial or financial relationships that could be construed as a potential conflict of interest.

## Publisher's Note

All claims expressed in this article are solely those of the authors and do not necessarily represent those of their affiliated organizations, or those of the publisher, the editors and the reviewers. Any product that may be evaluated in this article, or claim that may be made by its manufacturer, is not guaranteed or endorsed by the publisher.
